# The Experiences of Family Members of COVID-19 Patients in Iran: A Qualitative Study

**DOI:** 10.4314/ejhs.v31i6.4

**Published:** 2021-11

**Authors:** Elyas Hosseinzadeh Younesi, Zahra Sabzi, Mahbobeh Brojerdi, Shohreh Kolagari

**Affiliations:** 1 PhD candidate, Department of Nursing, Golestan University of Medical sciences, Gorgan, Iran; 2 Nursing Research Center, Golestan University of Medical Science, Gorgan, Iran; 3 Department of Geriatric Nursing, Golestan University of Medical Science, Gorgan, Iran

**Keywords:** SARS-CoV-2, Family, Patients, Qualitative Research

## Abstract

**Background:**

Being diagnosed with coronavirus disease-19 (COVID-19) usually causes emotional stress for patients and their families. Understanding the challenges faced by family members of COVID-19 patients is necessary to provide holistic family centered care to support patients and families on their journey to recovery from COVID-19. The aim of this study was to explore the experiences of Iranian family members of COVID-19 patients.

**Methods:**

This qualitative study was performed using the conventional content analysis approach in 2020 in Gorgan, northeastern Iran. Using a purposive sampling 15 family members of inpatient and outpatient COVID-19 patients who were involved in caring of their patients were selected. Data were collected using in-depth and semi-structured interviews and were analyzed by Graneheim and Lundman's content analysis method with the support of MAXQDA software.

**Results:**

Family members' experiences of COVID-19 patients were categorized into two main themes, four category and 10 subcategories. One of the main themes was “psychosocial distress”, with two categories of “uncertainty of the disease” and “perceived psychosocial burdens”. Another main theme was “adaptation strategies”, with the two sub-categories including “adaptative care” and “trusting God”.

**Conclusion:**

The results of this study provide a broad range of context-specific challenges faced by family members of Covid-19 patients. It is essential for healthcare providers to be aware of the complex psychological and social conditions of families of Covid-19 patients. Hence, healthcare managers and policymakers should implement preventive and supportive programs at all levels in hospitals and community and provide supportive strategies to reduce or eliminate their challenges.

## Introduction

The coronavirus disease-19 (COVID-19) pandemic poses a significant threat to the health of people and unique challenges to healthcare systems, worldwide (1–3). According to the World Health Organization, a total of 88,387,352 confirmed cases of COVID-19 had been identified around the world by January 12, 2021, out of which 1,919,204 deaths have occurred (4–5). It is expected that on average 1292 people are infected with COVID-19 daily in Iran (5–6). In addition to the progressive incidence and mortality rate of COVID-19 infection, this pandemic has devastating health, socio-economic and psycho-social consequences (7, 8). These conditions threaten the mental health of people in the community, the patients and their families (9–11).

Observance of prevention measures, disclosure of the disease to others, fear of the unknown future of the disease, fear of the death of loved ones, and quarantine can cause the family member of COVID-19 patients to lose their physical and mental balance due to anxiety, negative thoughts, and stress (13–16). In a cross-sectional study in China, a high prevalence of anxiety and self-reported depressive symptoms was reported among COVID-19 patients and their families (17). In another study, inadequate social support from family members, insufficient information, a reduction in family income, and the stigma of contracting infectious diseases was also reported in patients with COVID-19 (18). In addition, it has been shown that having a family member with COVID-19 can be associated with aggravates family conflicts, economic distress, violence, and epidemic tensions among family members (19).

Moreover, in Asian societies especially in Iranian culture and society, family is the primary support system for patients and family members usually have the most important role in the caring process of COVID-19 patients. Therefore, they may face some challenges (7, 17). It seems that using qualitative methods, can provide a deeper understanding of challenges experienced by family members of patients with COVID-19. While there is a growing body of literature on the experiences of COVID-19 patients as well as frontline healthcare workers caring for patients with COVID-19, there is limited evidence to evaluate the experience of family members of patients in Iranian COVID-19 literature. Therefore, the present qualitative study was conducted to explore the experiences of family members of COVID-19 patients in Iran.

## Methods

This qualitative study was performed using the conventional content analysis approach. Participants in this study were selected purposefully from the family members of COVID-19 patients who take care of their patients at home or take care of their COVID-19 patient in two referral hospitals of Sayad Shirazi and 5th Azar, affiliated to Golestan University of Medical Sciences in Gorgan, Golestan province, northeastern Iran.

Data were gathered through in-depth, semi-structured, face to face personal interviews with people who met the inclusion criteria. The inclusion criteria were older adults aged ≥20 years, family members of Covid-19 patients who have accepted responsibility for patient care in the hospital or at home, the ability to communicate in Persian, and the willingness and ability to share their experiences. Maximum variations in sampling in terms of age, sex, educational level, and occupation were considered during the participants' recruitment.

Interviews were performed at a date and time that was most convenient to participants. Interviews were guided by open-ended questions including “*describe your experiences of caring for a family member with COVID-19*”, “how you felt when your patient's COVID-19 test was positive?”, and “*what was the reaction of others when they saw you?*” The interviewer used probing questions such as “*can you give an example?*”, “*what do you mean? Can you explain more?*” to clarify a situation or to provide detail to an answer. Participants' nonverbal reactions were also recorded during the interview and used in the analysis. It was ended with a summary, the interview at this stage opening up for clarifying questions and final reflections. All interviews were conducted in Persian, by the first author, who is a PhD candidate with training and in conducting qualitative studies to ensure consistency.

All interviews were recorded verbatim and transcribed one by one in the first 24 hours. Also all field notes recorded in full immediately after each event and were analyzed. Data collection was continued up to data saturation, i.e. when the data became repetitive and no new data were obtained from the interviews and no new theme emerged. Although data saturation was achieved with thirteen interviews, two more interviews were held to ensure saturation. Eight interviews were conducted with the families of patients who were managed at home, and seven interviews with the families of patients admitted to the hospital. Interviews were performed from April to August 2020 and lasted between 40–60 minutes. Participants received no compensation for participating in the interview sessions.

Concurrently with data collection, data were analyzed using Graneheim and Lundman's six-step conventional content analysis approach (20). The analysis focused on the latent content which is the underlying meaning of the content which requires some levels of abstraction. First, immediately after each interview, it was transcribed word by word and the texts were reviewed and read several times to get a better understanding of the data and to accurately evaluate it. Second, the text was divided into meaning units that were condensed. Each meaning unit comprised words and sentences containing aspects related to each other. Third, the text was divided into meaning units and condensed, which is a constellation of words or statements related to the aim of the study. Fourth, the condensed meaning units were labeled with codes. Fifth, these codes were refined; that is, the similar codes were put together or merged. Thus, a number of subcategories and categories were formed and the hidden content and concepts were extracted. Finally, the underlying meaning and content of the data were extracted, and themes were formulated as the expression of the latent meaning of a text. The MAXQDA (Version 10) was used to organize, code, and manage data.

Trustworthiness was established using Guba and Lincoln's criteria (21). To ensure credibility, the researcher who collected the data and performed the analysis was continuously engaged with the data for five months in the research environment. Participants with a maximum variety were selected, and in-depth interviews were done to access rich data. Also, interviews' text and the related extractive codes were returned to four participants to ensure the accuracy of the codes. The peer-review method was used to improve confirmability. Two experts who did not participate in the study reviewed the data as well as the coding and classification procedures to ensure that the codes and categories were consistent with the data. To ensure data transferability, the researcher tried to present the implementation steps of the work to ensure data transferability so that the other researchers could judge the data transfer capability and follow the research processes. Finally, events and decisions related to various stages of the research, such as the interview and data analysis, were rigorously recorded to increase the data's dependability for review by others.

**Ethical considerations**: All participants were informed about the study aim, interview process, confidential data management, and voluntariness of participation. Participation in the present study was completely voluntary and informed consent was obtained from all participants. All of the interviews were audio taped with the consent of the participants. This study approved by the Research Ethics Committee of Golestan University of Medical Sciences (Ethics Code: IR.GOUMS.REC.1398.391).

## Results

In the present study, 15 participants were included. Of these, 10 (66.6%) were women, and 5 (33.4%) were men. 8 (53.3%) participants had a diploma or less, and 7 (46.6%) were housewives. Most participants (n=12, 80%) were spouses of the patients (Table 1). At the first stage of data analysis, 316 initial codes were extracted. After merging the primary codes, they were reduced to 223 based on similarities and differences. In total, two main themes, four category and 10 sub-categories were formed (Table 2).

**Table 1:** Demographic characteristic of the participants

Participants	Sex	Age	Level of Education	Occupation	Relationship with patient
P1	Female	51	Elementary	House Wife	**Bride**
P2	Female	43	Elementary	House Wife	**Spouse**
P3	Female	37	Bachelor	Clerk	**Spouse**
P4	Male	39	Bachelor	Clerk	**Spouse**
P5	Female	50	Bachelor	High school manager	**Spouse**
P6	Female	40	High School Degree	House Wife	**Daughter**
P7	Male	35	Bachelor	Clerk	**Spouse**
P8	Female	49	Elementary	House Wife	**Spouse**
P9	Male	44	Elementary	Self-Employment	**Spouse**
P10	Female	55	Illiterate	House Wife	**Spouse**
P11	Female	43	Associate	Degree Clerk	**Spouse**
P12	Male	56	Elementary	Self-Employment	**Spouse**
P13	Female	22	Undergraduate	Student	**Daughter**
P14	Female	38	High School Degree	House Wife	**Spouse**
P15	Male	35	Bachelor	Self-Employment	**Spouse**

**Table 2:** Main classes, secondary classes and sub classes related to the experiences of family members of COVID-19 patients

Theme	Category	Sub-category
**Psychosocial** **Distress**	Uncertainty of the disease	✓ The ambiguous nature and consequence of the disease
		✓ Dealing with inaccurate, scattered and contradictory information
	Perceived psychosocial burdens	○ Feeling of social isolation
		○ Perceived worries and fears
		○ Role conflict
**Adaptation** **Strategies**	Adaptive care	✓ Compassionate support
		✓ Trying to more learn
		✓ Denial of the disease
	Trusting God	○ Recourse unto God
		○ Commitment to religious beliefs

### 1. Psychosocial Distress

Two categories of “*Uncertainty of the disease*” and “*Perceived psychosocial problems*” was formulated into a theme as “*Psychosocial distress*”. This categorization was based on the similarity and appropriateness of the concept that expresses the mental and psychological difficulties of family members of Covid-19 patients.

#### 1.1. Uncertainty of the disease

The sub-categories of “*the ambiguous consequence of the disease*” and “*dealing with scattered and contradictory information*” are formulated into a more abstract concept as “*Uncertainty of the disease*”.

##### 1.1.1. The ambiguous nature and consequence of the disease

The lack of awareness about the ambiguous consequences of the Covid-19 disease was one of the most common concerns among the study's participants. A participant stated “*I was always thinking about what was going to happen. The doctors said that 60 percent of your husband's lungs were involved, but they did not say whether he would get better or not, whether he would survive or not*” (Participant 3).

##### 1.1.2. Dealing with inaccurate, scattered and contradictory information

Most families had shown different behaviors in dealing with the disease due to low awareness, lack of adequate health literacy, and receiving scattered and contradictory information about the disease.

#### 1.2. Perceived psychosocial burdens

The subcategories of “*feeling of social isolation*”, “*perceived worries and fears”, and “role conflict*” are categorized into a more abstract concept as “*perceived psychosocial burdens*”.

##### 1.2.1. Feeling of social isolation

Social isolation or feelings of social exclusion were also common contents among the participants.

“*Even now that my wife is fine, anyone who sees me on the street quickly leaves the place or moves away. People are still afraid of us and stay away from us”* (Participant 11).

##### 1-2-2- Perceived worries and fears

One of the common experiences expressed by most participants was the fear, worry, and anxiety caused by the disease for the caregiver, other family members, and the patient. “*During the disease course, my son was awake from 1 a.m. to 5 a.m. to ensure that nothing happened to his father. As soon as my son saw that his father's eyes close, he would quickly turn on the pulse oximeter to see how much oxygen was in his blood. He asked his father, “Are you awake? Are you sleep? How're you?” It was a very, very difficult time”* (Participant 8).

##### 1.2.2. Role conflict

Another experience expressed by most participants was the multiplicity of roles and sometimes the conflict between the roles of the caregiver. “*I work in the hospital's medical records department. On the one hand, all the leaves were canceled, and on the other hand, my husband wasn't feeling well, and he was afraid of being hospitalized. I had to go to work in the morning, and my husband was alone. He was always on my mind, and I didn't know what to do*” (Participant 3).

### 2. Adaptation Strategies

The categories of “adaptive care” and “trusting God” are formulated into a more abstract content as “adaptation strategies”.

#### 2.1. Adaptive care

The sub-categories of “compassionate support”, “trying to more learn”, and “denial of the disease” are categorized into a concept with maximum abstraction as “adaptive care”.

##### 2.1.1. Compassionate support

Most of the participants considered patient support as one of the most effective sources to promote hope in Covid-19 patients. They emphasized the importance of compassionate support in adapting and overcoming the challenges of this period.

##### 2.1.2. Trying to more learn

Participants' interviews indicated that they usually try to learn more as the golden circle of solving this period's challenges and as a strategy to adapt and overcome these problems. “*We used to ask others and conduct internet searches. I even paid 1,200,000 Rials when my daughter found a doctor on the internet who gave us advice on what to do. Because we were sick and we didn't know what to do, and we felt the need for a consultant*” (Participant 1).

##### 2.1.3. Denial of the disease

The majority of participants reacted with denial when a family member became ill or died due to COVID-19. Because the nature of the disease was frightening to people and affected them psychologically, feelings of panic, shock, and denial of the disease became more intense in people. “*My spouse advised us to wear gloves and masks when we went outside because the air was polluted. I didn't expect even one percent of this to happen (the participant starts crying). Because we weren't allowed to attend the funeral, I still can't believe I lost my husband*” (Participant 5).

#### 2.2. Trusting God

The sub-categories of “*recourse* unto God” and “*commitment to religious beliefs*” are categorized into more abstract contents as “*trusting God*”. Most families in their experiences have turned to God to reduce tension and increase their ability to overcome situations.

##### 2.2.1. Recourse unto God

Based on the experiences of most participants in the study, recourse to God can help caregivers and their patients in dealing with the disease in a positive, effective, and purposeful way by providing effective overcoming mechanisms like a defensive shield. “*I read the Qur'an or prayed when I was really tired and heartbroken*” (Participant 2).

##### 2.2.2. Commitment to religious beliefs

Belief in God and adherence to religious beliefs become more prominent and important in life crises, and people who faced life-threatening situations become more focused on their religious interests. In this study, most families of Covid-19 patients stated that adhere to religious beliefs gives them more power to fight the disease. “*I said to myself, this is a disease like all other viruses, so we could deal with it. I prayed to God, and it calmed me down in those circumstances*”. (Participant 14).

## Discussion

According to the perception of participants, the uncertainty of the disease can cause psychosocial distress in patients' families. In this regard, the model of intolerance of uncertainty and ambiguity, which is considered as a new perspective in explaining anxiety, states that anxious people often perceive vague and indefinite situations as disturbing and stressful (22). As a result, they suffer from psychosocial distress in the face of such circumstances (23). In line with the results of our study, other researchers confirmed that people's knowledge and understanding of symptoms, as well as observing the course and consequences of the disease, shape their emotional, behavioral, and cognitive reactions to the disease. These responses appear as feelings of fear, anxiety, and restlessness in the families of COVID-19 patients because of the experience of ambiguity, uncertainty, and dealing with scattered and contradictory information about the disease (24). In another study, facing ambiguous consequences of COVID-19 were mentioned as a reason for anxiety, stress, fear, and irrational thinking in people (25). Other studies in this field have found that facing uncertain conditions and ambiguous consequences of COVID-19 during their illness are the main factors in causing psychosocial disorders (26, 27). The results of a qualitative study which evaluated the experiences of family members of critically ill ventilated COVID-19 patients revealed that inability to feel connected to the patient and informed about care by family members can bring stress and uncertainty (28).

In this study, one of the main issues mentioned by the most of families was perceived psychosocial problems due to the ambiguous consequence of the disease, dealing with scattered and contradictory information, feeling of social isolation, perceived worries and fears, and role conflict. In general, all studies that have investigated the psychosocial problems of patients and their families during quarantine have reported major signs of psychological trauma such as emotional distress, fear, stress, anxiety, mood swings, isolation, conflict, and anger (29–30). Brooks et al. showed that shock, confusion, contradictory negative emotions, fear, and anxiety are the common psychological consequences of quarantine due to COVID-19 (31). Asgari et al. described the experience of people with COVID-19 as social isolation and exposure to negative attitudes of others, stigma, and social exclusion that was very difficult and challenging for patients and their families (32). In another study, Eisazadeh et al. consider the feeling of worthlessness for others, the feeling of forgetfulness, and rejection as the psychological consequences of COVID-19 patients and their families (33). Also, the results of another study in Iran revealed that fear and uncertainty about the future was a major change for in family members of a deceased COVID-19 patient (34). The experiences of family members of critically ill COVID-19 patients in France also indicate that they usually experienced feelings of powerlessness and unreality because of discontinuous and interrupted relationship with their loved one (35).

According to the results of this study, adaptive care and trusting God has been conceptualized as adaptation strategies. Adaptation strategies include efforts to tolerate, dominate, or minimize internal and external conflicts and demands that go beyond personal resources (36). The results of present study revealed that some individual strategies such as adaptive care through creating a suitable environment for the patient, providing a diet that is appropriate for the patient's condition, using complementary therapies, support and gaining more education by consulting with specialists and families with similar experiences, raising the patient's spirits with compassionate support, and the use of the defense mechanism of denial in the early stages are usually used for better coping with the challenges experienced by family members of patients with COVID-19. They found that families seeking more education and learning developed more preventative behaviors, and ultimately increased the well-being of their families (37). In addition, the participants in this study mentioned performing religious and spiritual rituals, recourse to God and the holy persons as adaptation strategies. In their study, Koenig et al. pointed to the importance of faith and religious obligations in reducing anxiety and strengthening the mind and body during the COVID-19 pandemic (38). In another study, religious activities were considered as an overcoming strategy to reduce anxiety and worry in patients with COVID-19 (30).

One of the strengths of the study is the qualitative design of the research to understand and find the depth of the participants' minds. A limitation of present study may be recall bias since participants addressed their current experience and sometimes previous experiences also. The lack of generalizability of the findings to all family members of COVID-19 patients in different geo-cultural contexts, due to small sample size and participants' socio-demographic and cultural characteristics, are another limitation of this study.

In conclusion, this study showed that the experiences of families of COVID-19 patients are associated with the ambiguous consequences of the disease, dealing with scattered and contradictory information, perceived worries and fear, feeling of social isolation, and role conflict. They used strategies to adapt and overcome psychosocial disorder such as compassionate support, trying for more learn, denial of disease, recourse, and adherence to religious observances. The findings of the research can help to better understand the issues, psychological needs, and health promotion, as well as how the family care process and can be used to increase the understanding and recognition of psychological problems and needs and promote the mental health of these families. Hence, health policymakers should implement preventive and supportive programs at all levels in the community and seek supportive strategies to reduce or eliminate problems.
